# Ultrasound-guided transversus abdominis plane (Tap) block catheterization for continuous analgesia in canine pancreatitis: Case report

**DOI:** 10.29374/2527-2179.bjvm005826

**Published:** 2026-11-05

**Authors:** Caio Henrique Gomes Freitas, Vitória Gonçalves Magalhães, Victor Duchi Chipolechi Quintanilha, Brenda Aparecida de Barros Kovacic

**Affiliations:** 1 UNIGRANRIO, RJ, Brazil; 2 Instituto de Veterinária (IV), Universidade Federal Rural do Rio de Janeiro (UFRRJ). Seropédica, RJ, Brazil; 3 Veterinarian Autonomus, Rio de Janeiro, RJ, Brazil; 4 Programa de Pós-Graduação em Medicina Veterinária (PPGMV), Departamento de Medicina e Cirurgia Veterinária, IV, UFRRJ. Seropédica, RJ, Brazil.

**Keywords:** interfascial block, Glasgow pain scale, perioperative medicine, regional analgesia, visceral pain, bloqueio interfascial, escala de dor de Glasgow, medicina perioperatória, analgesia regional, dor visceral

## Abstract

Acute pancreatitis in dogs is frequently accompanied by severe abdominal pain that may be refractory to conventional systemic analgesia. This report describes the case of an eight-year-old female Yorkshire Terrier with severe acute pancreatitis unresponsive to multimodal therapy with tramadol, dipyrone, and methadone. Due to persistent discomfort, ultrasound-guided bilateral transversus abdominis plane (TAP) block was performed using 0.125% bupivacaine (2 mg/kg) combined with dexamethasone (0.5 mg/kg), followed by interfascial catheter placement for intermittent administration every 8 hours over three days. Pain was objectively assessed using the Short Form of the Glasgow Composite Measure Pain Scale (CMPS-SF). The baseline pain score of 12/24 decreased markedly to 3/24 within the first hour post-procedure and remained consistently below 6/24 throughout hospitalization, eliminating the need for additional opioid rescue doses. The catheter was maintained under a semi-occlusive sterile dressing with no evidence of infection, leakage, or displacement. Continuous TAP block with perineural adjuvant therapy proved to be a feasible and promising multimodal strategy for managing somatic and referred pain in canine pancreatitis, although prospective, controlled clinical trials are required to elucidate its underlying mechanisms and efficacy.

## Resumo

A pancreatite aguda em cães é frequentemente acompanhada por dor abdominal intensa, que pode ser refratária à analgesia sistêmica convencional. Este relato descreve o caso de uma cadela da raça Yorkshire Terrier, com oito anos de idade, apresentando pancreatite aguda grave e sem resposta adequada à terapia multimodal com tramadol, dipirona e metadona. Devido à persistência do desconforto, foi realizado bloqueio bilateral do plano transverso do abdome (TAP), guiado por ultrassonografia, utilizando bupivacaína a 0,125% (2 mg/kg) associada à dexametasona (0,5 mg/kg), seguido do posicionamento de cateter no plano interfascial para administrações intermitentes a cada 8 horas durante três dias. A dor foi avaliada objetivamente por meio da forma abreviada da Escala Composta de Dor de Glasgow (*Short Form of the Glasgow Composite Measure Pain Scale* – CMPS-SF). O escore basal de dor, de 12/24, apresentou redução acentuada para 3/24 na primeira hora após o procedimento e permaneceu consistentemente abaixo de 6/24 durante toda a hospitalização, eliminando a necessidade de doses adicionais de resgate com opioides. O cateter foi mantido sob curativo estéril semioclusivo, sem evidências de infecção, extravasamento ou deslocamento. O bloqueio contínuo do TAP associado ao uso de adjuvante perineural mostrou-se uma estratégia multimodal viável e promissora para o manejo da dor somática e da dor referida em cães com pancreatite, embora estudos clínicos prospectivos e controlados sejam necessários para esclarecer seus mecanismos de ação e sua eficácia.

## Introduction

Pancreatitis is a relatively common disease in dogs and cats, occurring in acute or chronic forms with variable prognosis (Simpson, 2006; Weatherton & Streeter, 2009). The most frequent clinical signs include vomiting, diarrhea, anorexia, lethargy, and, notably, severe abdominal pain, which is considered a hallmark of the disease (Cardoso, 2015; Ferreira, 2014). In dogs, this pain is often manifested by the adoption of the "prayer position" (Costa Mack, 2020).

Therapeutic management relies on the triad of fluid therapy, nutritional support, and analgesia (Rondelli et al., 2009). Effective pain control is essential to promote comfort and therapeutic success, with opioids and other analgesic therapies being frequently administered (Cardoso, 2015; Mansfield, 2020).

## Case report

An eight-year-old female Yorkshire Terrier was presented to the veterinary service with vomiting, diarrhea, and acute abdominal pain. On initial physical examination, the patient was in lateral recumbency, lethargic, poorly responsive to stimuli, and showed marked discomfort upon abdominal palpation. Laboratory tests were performed, including complete blood count, urea, creatinine, ALT, alkaline phosphatase, total protein, and albumin, along with abdominal ultrasonography. Ultrasonographic examination revealed a hypoechoic and enlarged pancreas, accompanied by reactive adjacent mesentery (Figure 1). These findings were consistent with a diagnosis of acute pancreatitis (Hess et al., 1998; Kook et al., 2014).

**Figure 1:**
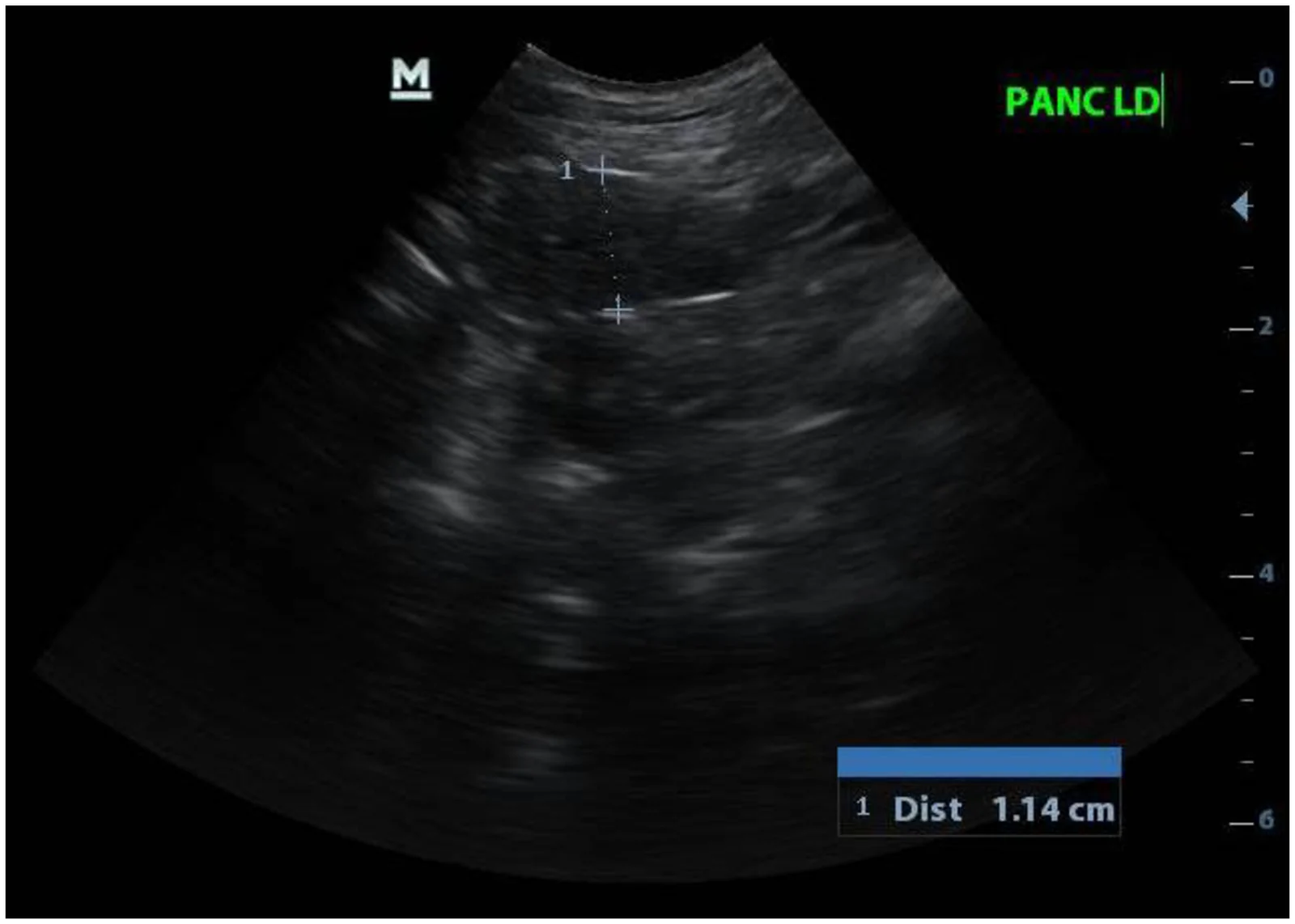
Ultrasonographic image of the right pancreatic lobe.

Pain scores were assessed using the Short Form of the Glasgow Composite Measure Pain Scale (CMPS-SF), which revealed a baseline score of 12/24, indicative of moderate-to-severe pain (Lima, 2024). Initial analgesic management consisted of tramadol (Tramadon Cristália Produtos Químicos Farmacêuticos Ltda., Brazil; 3 mg/kg) combined with dipyrone (Febrax LEMA Biologic do Brasil Ltda., Brazil; 25 mg/kg), followed by methadone (Mytedom Cristália Produtos Químicos Farmacêuticos Ltda., Brazil; 0.3 mg/kg). However, an adequate clinical response was not achieved.

Given the refractoriness to conventional analgesia and persistent discomfort, a transversus abdominis plane (*TAP*) block was chosen as an adjuvant technique for pain management. To perform the block, the patient was sedated with propofol (Propovan Cristália Produtos Químicos Farmacêuticos Ltda., Brazil; 4 mg/kg), titrated to clinical effect. Following sedation, clipping of the thoracolumbar region from T11 to L3 was performed, followed by surgical skin antisepsis with 2% chlorhexidine scrub and 70% alcohol. For the ultrasound-guided block, 0.125% bupivacaine (Bupivacaína Cristália Produtos Químicos Farmacêuticos Ltda., Brazil; 2 mg/kg) was combined with dexamethasone (Dexamethasone Disodium Phosphate Teuto Brasileiro S/A, Brazil; 0.5 mg/kg). A portable ultrasound system (SonoEye P2 VET; Chison) equipped with a 7.5 to 12 MHz transducer was used.

With the patient positioned in lateral recumbency and the target side facing upward, the probe was positioned transversely across the body midline, between the iliac crest and the last rib. A Tuohy needle attached to a 5 mL syringe was advanced in-plane until it reached the fascial plane between the internal oblique and transversus abdominis muscles. Visualization of interfascial plane hydrodissection confirmed correct needle placement. For continuous local anesthetic delivery, a 20G catheter was introduced into the hydrodissected interfascial plane. The catheter was secured using the Roman sandal ("ballerina") suture technique with 2-0 nylon (Figure 2). After completing the procedure on the initial side, the patient was repositioned to perform the block on the contralateral hemiabdomen.

**Figure 2:**
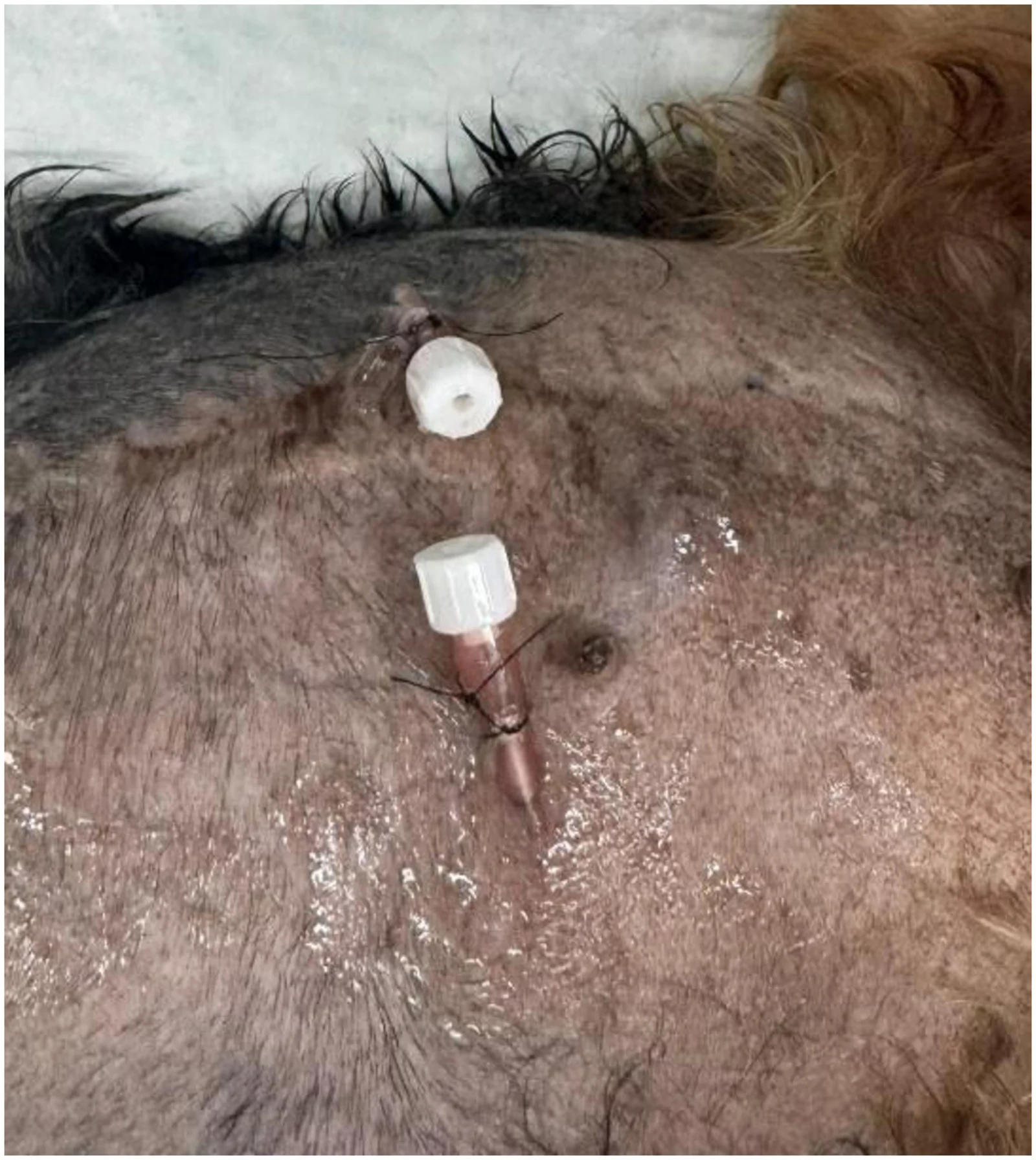
20G catheter positioned and secured bilaterally to allow for continuous administration of local anesthetic.

Pain was assessed using the CMPS-SF scale hourly during the first 6 hours, followed by evaluations at 8 and 12 hours post-block and subsequently every 6 hours until hospital discharge. The rescue analgesia protocol consisted of intravenous methadone (0.2 mg/kg) if the pain score exceeded 6 points. Analgesia was maintained exclusively via the interfascial catheter through administration of 0.125% bupivacaine (2 mg/kg) every 8 hours, combined with dexamethasone (0.5 mg/kg) on the first and final days. Prior to local anesthetic administration, catheter hubs were disinfected with 2% chlorhexidine scrub and 0.5% chlorhexidine solution. In addition, the insertion site was maintained under a sterile, semi-occlusive dressing, which was changed every 12 hours; no signs of local infection, anesthetic leakage, or catheter displacement were observed. The analgesic catheter remained in place for 3 days and was removed 8 hours prior to patient discharge for home recovery.

## Discussion

Pain is a complex sensory perception resulting from the activation of nociceptors located in somatic and visceral tissues (Reece, 2017). In visceral pain, such as that associated with the pancreas, nociceptors consist of unmyelinated free nerve endings sensitive to distension, stretching, inflammation, and ischemia. Nociceptive signals are transduced into action potentials and travel via visceral sensory sympathetic fibers. These fibers enter the spinal cord through white rami communicantes, synapse in the dorsal horn, and ascend via the spinothalamic tract to the thalamus and somatosensory cortex, where pain perception occurs (Ferrez, 2020). Understanding this neuroanatomy is essential, as visceral pain in pancreatitis is typically diffuse and poorly localized. This characteristic stems from the convergence of visceral and somatic afferent inputs onto the same second-order neurons in the dorsal horn, producing referred pain perceived in superficial cutaneous or muscular regions (Ferrez, 2020).

In acute pancreatitis, this pain is often referred to the epigastric region and flanks, explaining classical antalgic postures such as the "prayer position," in which the dog lowers its cranial torso while keeping the pelvic limbs extended to relieve abdominal pressure (Pessôa et al., 2004). Autonomic innervation to the pancreas originates predominantly from the splanchnic nerves (T5–T12 and L1–L2) converging onto the celiac and cranial mesenteric plexuses, corroborating the widespread and systemic character of pancreatic pain (Cima, 2020; Ghoshal, 1975; Rocha & Massone, 2006).

In this setting, the transversus abdominis plane (*TAP*) block emerges as a valuable component of multimodal analgesia. The *TAP* block provides desensitization of the ventral and lateral abdominal wall, including skin, musculature, and parietal peritoneum. The ultrasound-guided approach deposits local anesthetic into the fascial plane between the internal oblique and transversus abdominis muscles. In this case, 0.125% bupivacaine at 2 mg/kg was utilized. According to Zoff et al. (2017) and Bruggink et al. (2012), adequate injection volume promotes cranial-to-caudal spread, blocking the ventral rami of spinal nerves T11 to L3, conferring broad abdominal wall analgesia. During real-time ultrasonography, relevant anatomical landmarks were identified and fascial hydrodissection was visualized without technical complications.

The first postoperative pain assessment was conducted 30 minutes following the procedure via CMPS-SF to exclude residual sedative effects from propofol that could confound scoring. After the initial 12 hours, monitoring proceeded every 6 hours until discharge. It is well established that, as a fascial plane block, the *TAP* block does not directly block deep autonomic visceral plexuses (Gambim, 2022; Portela et al., 2018). However, accurate volume deposition alleviates the somatic component of referred pain and modulates spinal central sensitization (Portela et al., 2018). In this context, Serighelli Júnior et al. (2024) reported the successful application of a continuous *TAP* block combined with methadone for postoperative analgesia following pancreatectomy in a dog. Freitag et al. (2018) investigated continuous *TAP* catheterization in two dogs with acute pancreatitis and one dog undergoing exploratory laparotomy, in which continuous 0.5% bupivacaine infusion provided satisfactory analgesia without rescue interventions. According to Freitag et al. (2018), one patient with pancreatitis showed a reduction in Glasgow pain score from a baseline of 15/24 to 5/24 and 1/24 following continuous catheter infusion. Similarly, the patient in this report showed a substantial reduction from a baseline score of 12/24 to 3/24 within the first hour post-intervention, maintaining low scores throughout hospitalization without opioid rescue requirements (Lima, 2024). These findings suggest that somatic analgesia provided by continuous *TAP* block can modulate referred pain and parietal hyperalgesia in canine pancreatitis.

Furthermore, the addition of dexamethasone as a perineural adjuvant may have extended analgesic duration. According to a Cochrane systematic review (Pehora et al., 2017), perineural dexamethasone significantly prolongs local anesthetic duration through local anti-inflammatory actions, attenuated systemic absorption, and direct modulation of nociceptive fiber excitability. Human meta-analyses (Chen et al., 2018; Zhang et al., 2019) have shown that dexamethasone in *TAP* blocks reduces pain scores, opioid requirements, and postoperative nausea and vomiting. While the favorable clinical course in this patient coincided with perineural dexamethasone addition, veterinary clinical trials evaluating dexamethasone adjuvants in *TAP* blocks remain limited. Pharmacologically, extrapolating human data appears rational given dexamethasone’s local anti-inflammatory and membrane-stabilizing effects; nonetheless, targeted clinical trials are warranted to clarify its specific contribution in animal continuous plane blocks.

## Conclusion

Ultrasound-guided transversus abdominis plane catheterization for intermittent local anesthetic infusion combined with dexamethasone was demonstrated to be a feasible and promising multimodal strategy for pain management in a dog with acute pancreatitis. This intervention was associated with an immediate reduction in clinical pain scores and no requirement for opioid rescue analgesia during hospitalization. As this is a single case report, the precise analgesic mechanisms and relative contributions cannot be definitively determined; therefore, prospective controlled clinical trials are essential to validate the efficacy and indications of interfascial wall blocks in modulating pancreatic pain in dogs.
